# ﻿Description of two new huntsman spiders from Vietnam (Araneae, Sparassidae)

**DOI:** 10.3897/zookeys.1236.145146

**Published:** 2025-04-29

**Authors:** He Zhang, Quang Duy Hoang, Shuang Lei

**Affiliations:** 1 Guo Shoujing Innovation College, Xingtai University, Xingtai City 054001, Hebei Province, China Xingtai University Xingtai City China; 2 Hebei Province Sweet Potato Breeding and Application Technology Innovation Center, Xingtai City 054001, Hebei Province, China Hebei Province Sweet Potato Breeding and Application Technology Innovation Center Xingtai City China; 3 Arachnid Resource Centre of Hubei & Hubei Key Laboratory of Regional Development and Environmental Response, Faculty of Resources and Environmental Sciences, Hubei University, Wuhan City 430062, Hubei Province, China Hubei University Wuhan China; 4 Tay Nguyen University, 567 Le Duan, Buon Ma Thuot City 630000, Dak Lak Province, Vietnam Tay Nguyen University Buon Ma Thuot City Vietnam; 5 Shengzhou Agricultural Technology Extension Center, Shengzhou City 312400, Zhejiang Province, China Shengzhou Agricultural Technology Extension Center Shengzhou City China

**Keywords:** Biodiversity, *Heteropoda*, huntsman spider, new species, *Pseudopoda*, sparassid systematics, taxonomy, Vietnam

## Abstract

Two new species of the sparassid genera *Heteropoda* Latreille, 1804 and *Pseudopoda* Jäger, 2000 are described from Vietnam: *Heteropodataygiangensis***sp. nov.** (♂) from Quang Nam Province and *Pseudopodatadungensis***sp. nov.** (♀) from Dak Nong Province. The new *Pseudopoda* species is described and diagnosed based on both morphological characteristics and DNA barcoding. DNA barcode data (COI) are provided for both new species.

## ﻿Introduction

The family Sparassidae Bertkau, 1872, commonly known as huntsman spiders, is the tenth largest spider family globally, comprising 1531 valid species in 98 genera (World Spider Catalog 2025). Recently, several studies have focused on the family in Vietnam (Logunov and Jäger 2015; Zhang et al. 2023; Korai and Jäger 2024a). However, only 28 species of huntsman spiders have been documented, belonging to the following genera: *Heteropoda* Latreille, 1804 (11 species); *Pandercetes* L. Koch, 1875 (1 species); *Pseudopoda* Jäger, 2000 (9 species); *Rhitymna* Simon, 1897 (2 species); *Sinopoda* Jäger, 1999 (4 species); and *Thelcticopis* Karsch, 1884 (1 species) (World Spider Catalog 2025). Clearly, research on Vietnamese huntsman spiders remains limited, and the relatively low diversity reported may reflect an artifact due to insufficient taxonomic studies rather than reflecting the true species richness of the region.

This study aims to enhance the taxonomic understanding of the huntsman spiders in Vietnam by describing two new species: *Heteropodataygiangensis* sp. nov. from central Vietnam and *Pseudopodatadungensis* sp. nov. from the central highlands of Vietnam. These genera are among the most diverse in the region, and the new species described here will contribute to a more accurate representation of the huntsman spider fauna in Vietnam.

## ﻿Material and methods

All specimens were collected by hand and examined with an Olympus SZX16 stereomicroscope; details were further investigated with an Olympus BX51 compound microscope. Colouration is described in all species from specimens in ethanol. Copulatory organs were examined and illustrated after dissection from the spider bodies; epigynes were cleared with Proteinase K. Habitus photos were obtained using a Leica M205 C digital microscope attached to a Leica DMC4500 digital camera. Coordinates are given in square brackets when retrieved secondarily from Google Earth.

Leg measurements are shown as: total length (femur, patella, tibia, metatarsus, tarsus). Number of spines is listed for each segment in the following order: prolateral, dorsal, retrolateral, ventral (in femora and patellae ventral spines are absent and the fourth digit is omitted in the spination formula). The terminology used in text and figure legends follows Li et al. (2013) and Quan et al. (2014). All measurements are given in millimetres. The map was produced using ArcMap ver. 10.8.1.

All specimens treated in the present paper were compared with individuals of described species within a certain distribution range to avoid describing synonyms.

Abbreviations used in text and figures: **AB**, anterior bands; **ALE**, anterior lateral eyes; **AME**, anterior median eyes; **C**, conductor; **CH**, clypeus height; **CO**, copulatory opening; **dRTA**, dorsal part of retrolateral tibial apophysis; **DS**, dorsal shield of prosoma; **E**, embolus; **FD**, fertilization duct; **Fe**, femur; **IDS**, internal duct system; **LL**, lateral lobes; **MHU**, Museum of Hubei University, Wuhan, China; **Mt**, metatarsus; **OL**, opisthosoma length; **OS**, opisthosoma; **OW**, opisthosoma width; **Pa**, patella; **PL**, prosoma length; **PLE**, posterior lateral eyes; **PME**, posterior median eyes; **Pp**, palp; **PW**, prosoma width; **RTA**, retrolateral tibial apophysis; **S**, spermophor; **Ti**, tibia; **TL**, total length; **vRTA**, ventral part of retrolateral tibial apophysis; **I**, **II**, **III**, **IV**, legs I to IV.

To obtain DNA barcodes, a mitochondrial gene (mitochondrial cytochrome *c* oxidase subunit I, COI) was amplified and sequenced from two specimens. DNA extraction, PCR amplification, and sequencing followed the protocols described by Zhang et al. (2021). The universal primers LCO1490 and HCO2198 (Folmer et al. 1994) were used for PCR amplification. GenBank accession numbers for the newly generated sequences are listed in Table 1.

**Table 1.** Information on newly sequenced *Heteropodataygiangensis* sp. nov. (paratype) and *Pseudopodatadungensis* sp. nov. (holotype) with specimen label and GenBank accession numbers.

Species name	Voucher code	Accession number
*Heteropodataygiangensis* sp. nov.	LJ20240069	PV426987
*Pseudopodatadungensis* sp. nov.	LJ20240005	PV426988

For phylogenetic analysis, the newly obtained sequence of *Pseudopodatadungensis* sp. nov. was incorporated into the COI dataset from Cao et al. (2016), while the sequence of *Heteropodataygiangensis* sp. nov. was incorporated into a COI dataset retrieved from GenBank. Phylogenetic analyses were performed following the methodologies outlined in Cao et al. (2016) and Zhang et al. (2021).

## ﻿Result

### ﻿Taxonomy

**Family Sparassidae Bertkau, 1872**

**Subfamily Heteropodinae Thorell, 1873**

#### ﻿ Heteropoda taygiangensis sp. nov.

Taxon classificationAnimaliaAraneaeSparassidae

61BD7540-B3F0-5828-9BE7-FA355F1BC64A

https://zoobank.org/A6319416-85FF-430E-9ED3-7155AD8343A6

Figs 1 , 2 , 3 , 7

##### Type material.

***Holotype*** male: Vietnam • Quang Nam Province: Tay Giang District, 15.8377°N, 107.3819°E, elevation 1353 m, 23 July 2019, Quang Duy Hoang leg. (MHU, LJ20240068). ***Paratype***: • 1 male, same locality data as holotype, collected on 18 July 2024, Quang Duy Hoang leg. (MHU, LJ20240069).

##### Etymology.

The specific name is derived from the type locality, the Tay Giang District; adjective.

##### Diagnosis.

**Figure 1.**
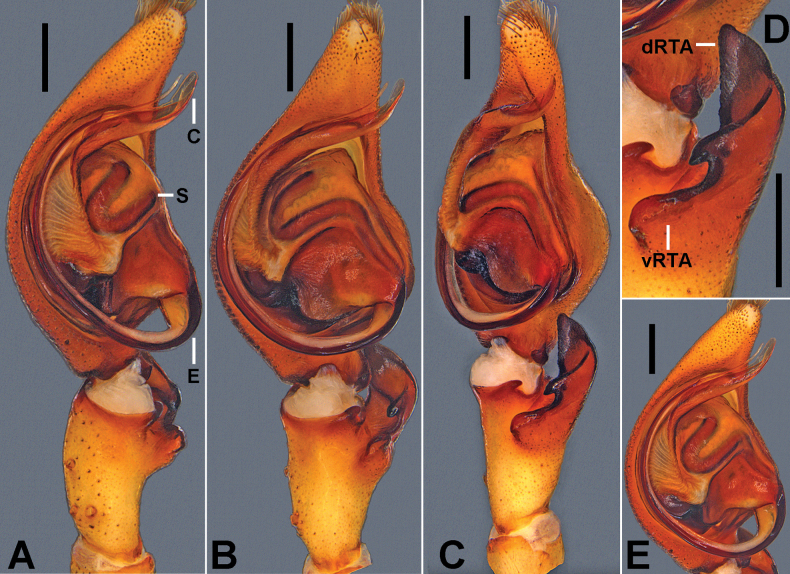
*Heteropodataygiangensis* sp. nov., holotype, male **A–C** left male palp (**A** prolateral **B** ventral **C** retrolateral) **D** left male palpal tibia, retrolateral **E** left male palpal cymbium, retrolateral. Abbreviations: C, conductor; dRTA, dorsal retrolateral tibial apophysis; E, embolus; S, spermophor; vRTA, ventral retrolateral tibial apophysis. Scale bars: 0.5 mm.

Males of *H.taygiangensis* sp. nov. resemble that of *H.hainanensis* Korai & Jäger, 2024 in having a similar shape of the tegulum and embolus, but can be distinguished from the latter by: (1) Conductor long, narrow, and distally curved in ventral view (Fig. 1A–C, E) (broader and slightly more rounded distally in *H.hainanensis*, see fig. 5A in Korai and Jäger 2024b); (2) spermophor slightly curved in distal tegular half (Fig. 1B) (obviously bent in distal tegular half in *H.hainanensis*, see fig. 5A in Korai and Jäger 2024b); and (3) dRTA significantly wide, bent inward, and blunt-tipped, presenting a short and pointed projection, directed ventrally (Fig. 1B–D) (narrow, bent outward and pointed tip, without any projection in *H.hainanensis*, Korai and Jäger 2024b: figs 5A, 6B).

##### Description.

**Male (*holotype*)**: Measurements: Medium-sized. TL 10.4, PL 5.6, PW 5.2, OL 4.8, OW 3.2. Eyes: AME 0.25, ALE 0.45, PME 0.31, PLE 0.47, AME–AME 0.20, AME–ALE 0.14, PME–PME 0.37, PME–PLE 0.25, AME–PME 0.27, ALE–PLE 0.42, CHAME 0.44, CHALE 0.33. Spination: Pp 131, 100, 2121; Fe I 123, II–III 323, IV 33(4)1; Pa I–II 101, III–IV 001; Ti I–IV 2226, III–IV 2126; Mt I–II 1014, III 2014, IV 3036. Measurements of palps and legs: Palp 7.5 (3.0, 1.6, 0.7, -, 2.2); I 22.5 (5.9, 2.4, 6.4, 5.8, 2.0); II 24.2 (6.5, 2.6, 7.3, 6.1, 1.7); III 18.5 (5.0, 2.2, 5.3, 4.8, 1.2); IV 20.2 (5.4, 1.8, 5.5, 5.6, 1.9). Leg formula: II-I-IV-III. Cheliceral furrow with 3 promarginal, 4 retromarginal teeth and ca. 42 denticles.

***Palp*** (Fig. 1A–E): As in diagnosis. RTA arising distally on tibia, vRTA in retrolateral view with rounded hump. Cymbium almost two times as long as Ti. Conductor long, beyond cymbial margin. Spermophor strongly curved close to the base of conductor. Embolus arising from tegulum in 4:00 o’ clock position, filiform and almost forming a semicircular in ventral view.

**Figure 2.**
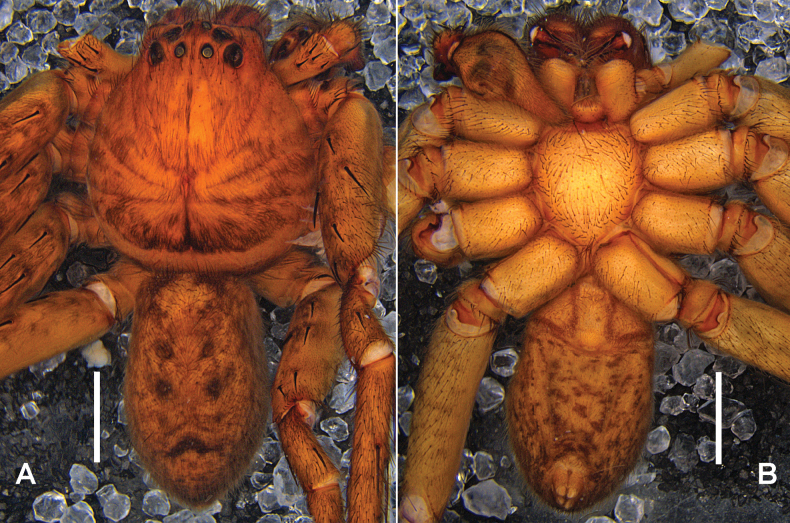
*Heteropodataygiangensis* sp. nov. **A, B** male habitus (**A** dorsal **B** ventral). Scale bars: 2 mm.

***Colouration*** (Fig. 2A, B): DS reddish brown, with dense black hairs in the posterior part. Fovea and radial marks distinct. OS dorsally with four dark round marks, regularly arranged in the median field, and with a dark transverse line in posterior part. OS ventrally yellow, with lots of reddish-brown spots, irregularly arranged.

**Female**: Unknown.

##### Distribution.

Known only from the type locality (Fig. 7).

##### Notes.

**Figure 3.**
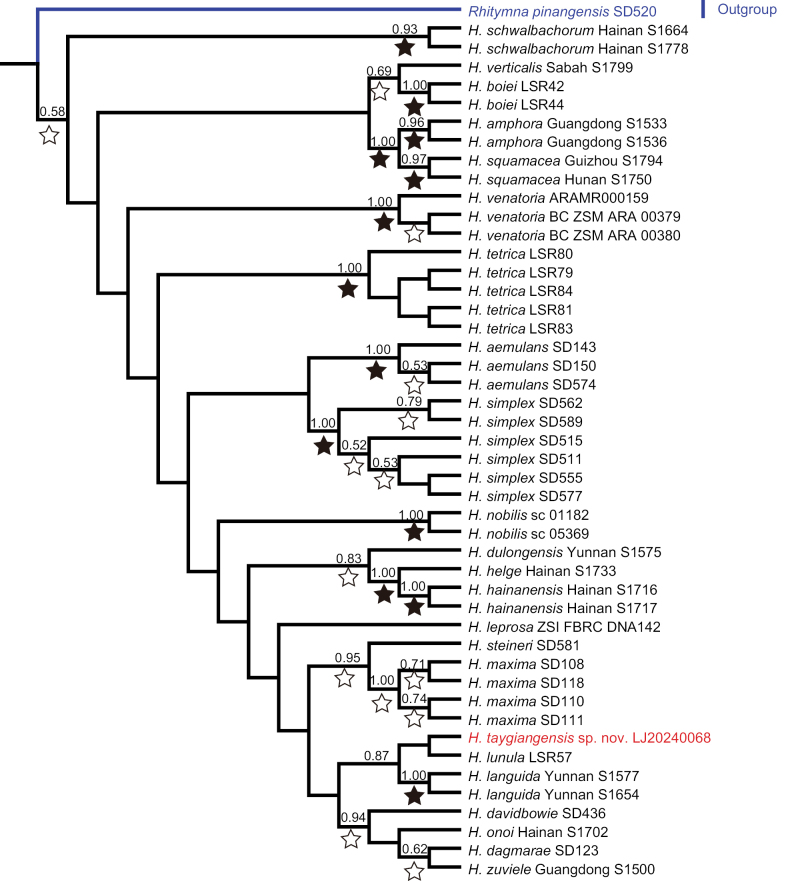
Bayesian tree based on the COI dataset including 46 *Heteropoda* individuals belonging to 23 species. Numbers on nodes are posterior probabilities; bootstrap support from ML analyses is indicated as solid stars for values > 95%, open stars > 50–95%. Red font indicates *H.taygiangensis* sp. nov., blue line indicates the outgroup.

To date, four *Heteropoda* species from Vietnam are known only from females: *H.altmannae* Jäger, 2008 from Ca Mau Province in southern Vietnam; *H.ignichelis* (Simon, 1880) from Sai Gon, southern Vietnam; *H.pressula* Simon, 1886 from Bachiou (=Ba Chieu?), Sai Gon, Vietnam; and *H.zuviele* Jäger, 2008 from northern Vietnam (Simon 1880, 1886; Jäger 2008). The newly described species, *H.taygiangensis* sp. nov., is represented by male specimens and could potentially be conspecific with one of these species, which are known only from females. However, *H.taygiangensis* sp. nov. is found more than 500 km away from the known localities of *H.altmannae*, *H.ignichelis*, and *H.zuviele*. *Heteropodaaltmannae* and *H.ignichelis* have only been recorded from their original localities, with no further records available. Although *H.zuviele* has been documented beyond its type locality, all known records are restricted to the north of the type locality. The type locality of *H.pressula*, “Bachiou,” remains uncertain. Simon (1886) repeatedly referred to it as a locality in Cambodia, but its exact location has not been verified. Because “Bachiou” cannot be confidently located in Cambodia, some subsequent authors (Ono et al. 2012; Yamasaki et al. 2018; Seyfulina and Kartsev 2022) have suggested it may correspond to Ba Chieu, Sai Gon, Vietnam. Determining whether “Bachiou” lies in Vietnam or Cambodia requires further investigation, particularly through re-examination of the *H.pressula* type specimen. Therefore, we consider the new species, *H.taygiangensis* sp. nov. to be a separate species in the present study. To support future efforts to resolve this taxonomic ambiguity, we provide COI sequence data. Additionally, we constructed the first phylogenetic tree of the genus *Heteropoda* based on all currently available COI sequences (Fig. 3).

#### ﻿ Pseudopoda tadungensis sp. nov.

Taxon classificationAnimaliaAraneaeSparassidae

7A5DF956-30B9-59BE-B2AB-DB955DC1392F

https://zoobank.org/EB77EE59-7A7C-4A9F-8342-CFED94C2AAA1

Figs 4 , 5 , 6 , 7

##### Type material.

***Holotype*** female: Vietnam • Dak Nong Province: Dak Glong District, Ta Dung National Park, 11.8616°N, 107.9923°E, elevation 953 m, 1 February 2024, Quang Duy Hoang leg. (MHU, LJ20240005).

##### Etymology.

The specific name is derived from the type locality, the Ta Dung National Park; adjective.

##### Diagnosis.

The female of *P.tadungensis* sp. nov. can be distinguished from all congeners by the medially incompletely fused lateral lobes in ventral view, and the internal duct system twisted into a wing shape.

##### Description.

**Female (*holotype*)**: Measurements: Medium-sized. TL 10.1, PL 4.4, PW 4.0, OL 5.7, OW 2.7. Eyes: AME 0.14, ALE 0.24, PME 0.17, PLE 0.23, AME–AME 0.16, AME–ALE 0.09, PME–PME 0.20, PME–PLE 0.35, AME–PME 0.27, ALE–PLE 0.25, CHAME 0.37, CHALE 0.30. Spination: Pp 131, 101, 2101, 1014; Fe I 323, II–III 322, IV 331; Pa I–III 101, IV 000; Ti I–II 2228, III 2226, IV 2126; Mt I–II 3034, III–IV 3036. Measurements of palps and legs: Pp 7.6 (1.6, 0.7, 1.4, –, 2.3); I 19.3 (5.5, 1.9, 5.5, 5.0, 1.4); II 22.0 (6.1, 2.2, 6.4, 5.6, 1.7); III 16.0 (4.9, 1.7, 4.4, 4.0, 1.0); IV 18.8 (5.6, 1.1, 5.1, 5.5, 1.5). Leg formula: II-I-IV-III. Cheliceral furrow with ca. 20 denticles.

**Figure 4.**
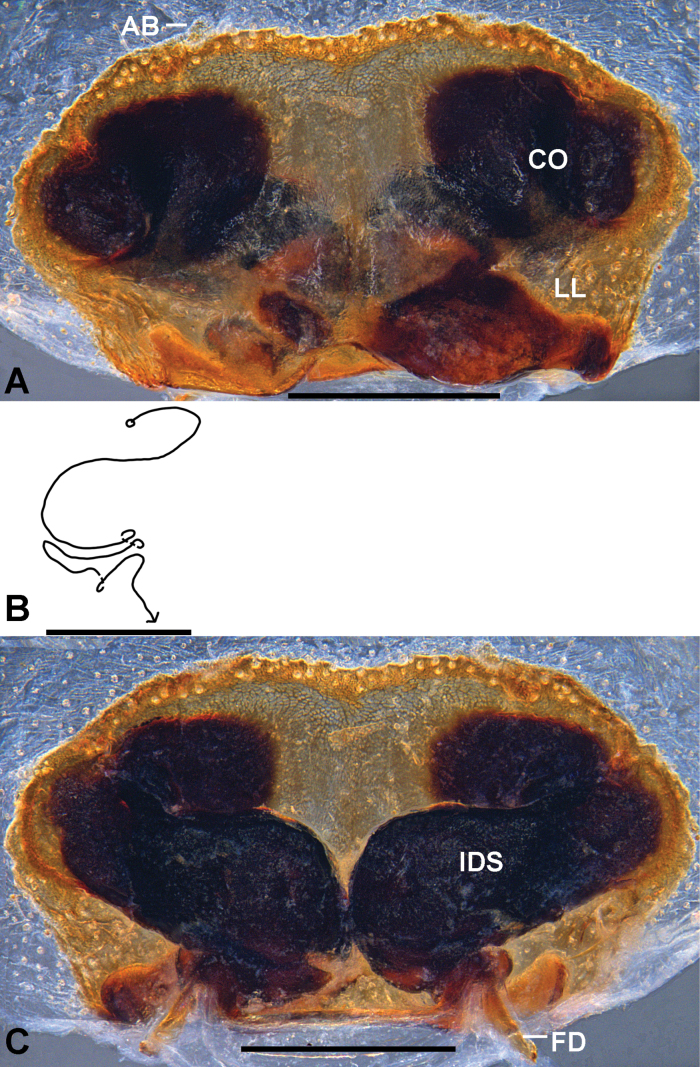
*Pseudopodatadungensis* sp. nov., holotype, female **A** epigyne, ventral **B** schematic course of IDS, dorsal **C** vulva, dorsal. Abbreviations: AB, anterior bands; CO, copulatory opening; FD, fertilization duct; IDS, internal duct system; LL, lateral lobes. Scale bars: 0.5 mm.

***Epigyne*** (Fig. 4A–C): As in diagnosis. Epigynal field wider than long. Anterior bands short. Anterior margins of lateral lobes slightly curved, posterior margins of lateral lobes with median indentation. Posterior part of internal duct system with loops. Fertilization duct long and narrow, situated postero-laterally.

**Figure 5.**
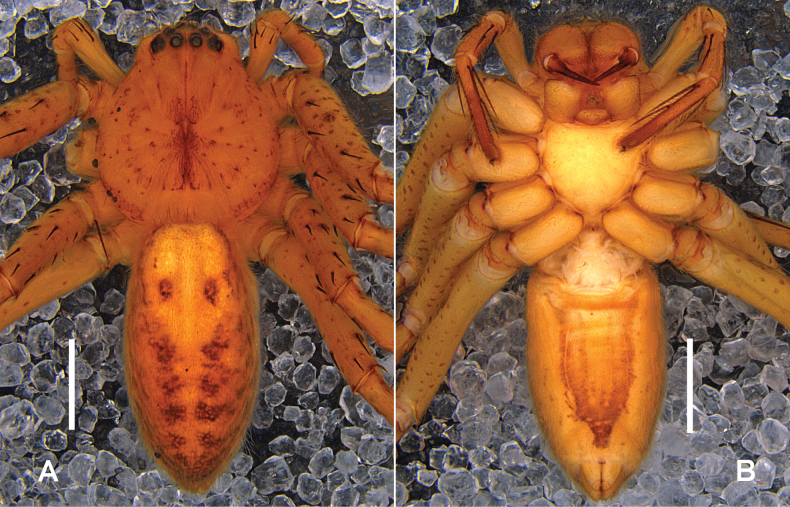
*Pseudopodatadungensis* sp. nov. **A, B** female habitus (**A** dorsal **B** ventral). Scale bars: 2 mm.

***Colouration*** (Fig. 5A, B): DS reddish brown, with dark spots. Fovea and radial marks distinct. OS dorsally with reddish-brown patches, arranged symmetrically in two longitudinal lines. OS ventrally yellow with a longitudinal brown region, margins with few small marks.

**Male**: Unknown.

**Distribution.** Known only from the type locality (Fig. 7).

##### Note.

**Figure 6.**
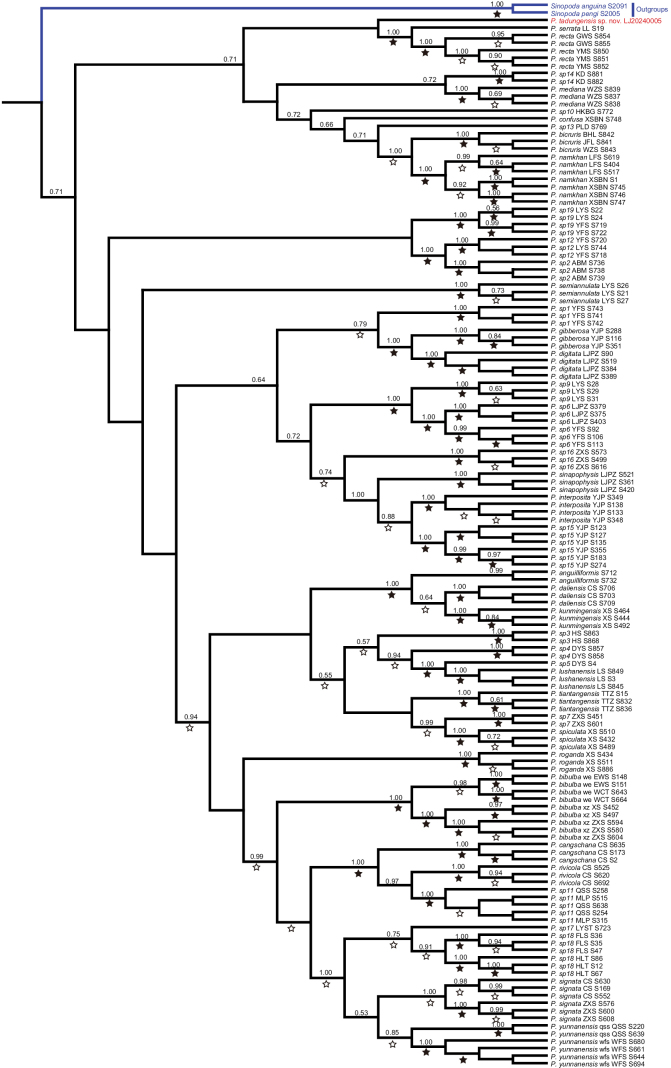
Bayesian tree based on the COI dataset including 139 *Pseudopoda* individuals belonging to 42 species. Numbers on nodes are posterior probabilities; bootstrap support from ML analyses is indicated as solid stars for values > 95%, open stars > 50–95%. Red font indicates *P.tadungensis* sp. nov., blue lines indicate the outgroup clade.

*Pseudopodatadungensis* sp. nov. exhibits subtle differences in genital morphology compared to most other species of the genus. To confirm its generic placement, we amplified the COI sequence of the holotype and conducted a phylogenetic analysis based on currently available COI sequences of the genus. The resulting tree supports the placement of *P.tadungensis* sp. nov. within *Pseudopoda* (Fig. 6). These molecular data provide an additional line of evidence for the validity and generic assignment of the new species.

Up to now, four *Pseudopoda* species from Vietnam are known only from males. The collection sites of these four species are located at a considerable distance from *P.tadungensis* sp. nov. (all exceeding 200 km, outside the endemic range of most *Pseudopoda* species; personal observation, Zhang et al. 2023), and their size and patterns do not match. Therefore, *P.tadungensis* sp. nov. is currently regarded as a distinct species. Further studies are required to address this ambiguity conclusively.

**Figure 7.**
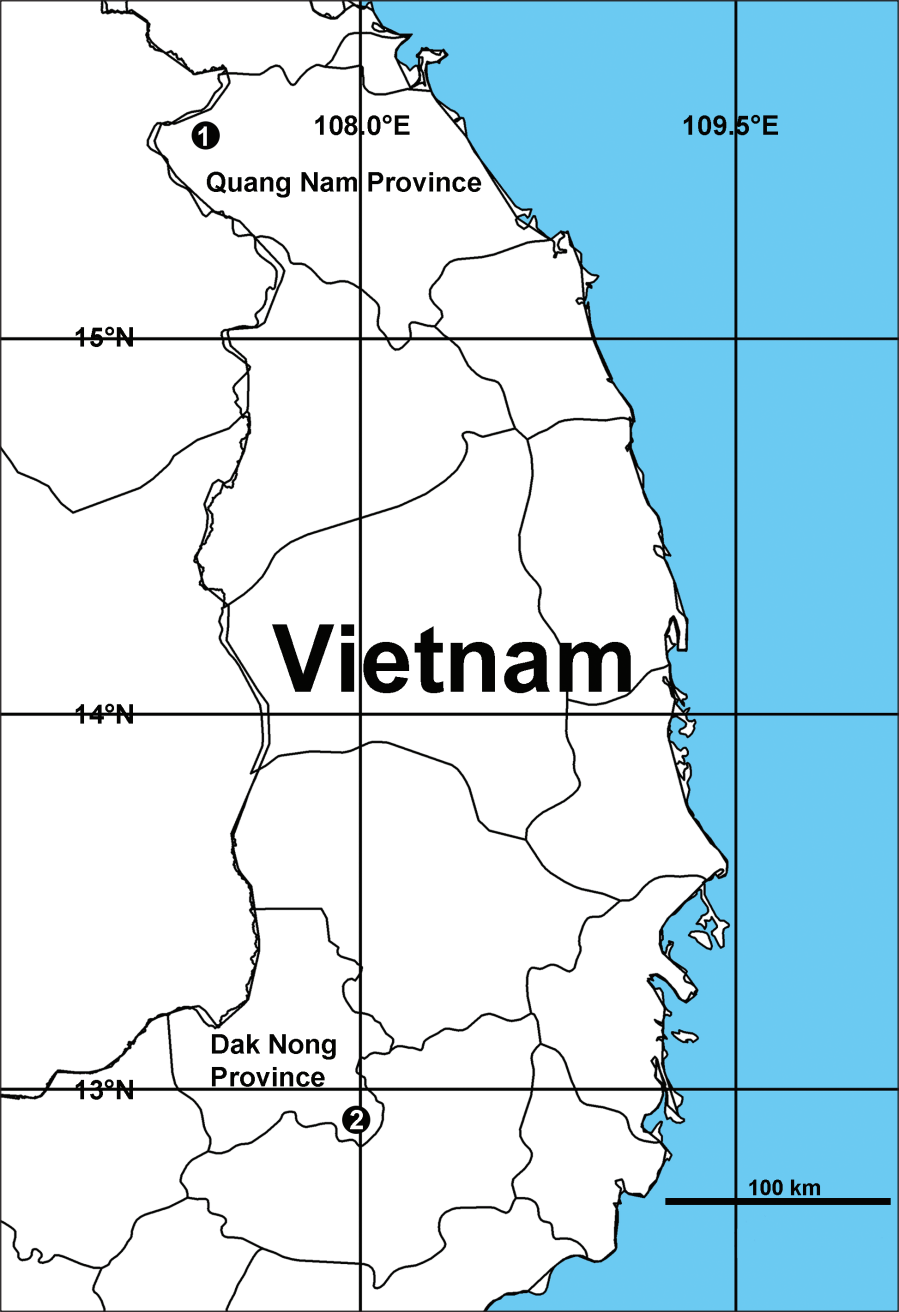
Records of the new *Heteropoda* and *Pseudopoda* species in Vietnam. 1 *H.taygiangensis* sp. nov. 2 *P.tadungensis* sp. nov.

## ﻿Discussion

The genera *Pseudopoda* Jäger, 2000 and *Heteropoda* Latreille, 1804 are among the most species-rich lineages within Sparassidae. However, the faunal diversity of both genera in Vietnam remains poorly documented, and recent surveys continue to uncover new taxa through targeted fieldwork and integrative taxonomic approaches.

In the present study, two new species, *P.tadungensis* sp. nov. and *H.taygiangensis* sp. nov., are described based on detailed morphological examinations. To further validate their taxonomic placements, COI barcoding and phylogenetic analyses were conducted as independent molecular evidence.

In the *Heteropoda* phylogeny (Fig. 3), *H.taygiangensis* sp. nov. clusters with other representatives of the genus, including *H.languida* and *H.lunula*, with strong support (posterior probability = 0.87). This finding agrees with the observed morphological characteristics and further supports placing it in the genus *Heteropoda*.

Similarly, in the COI-based phylogenetic tree (Fig. 6), *P.tadungensis* sp. nov. is recovered within the *Pseudopoda* clade and forms a well-supported monophyletic group (posterior probability = 0.73), confirming its placement in the genus. Although the species differs from most congeners by having fused lateral lobes (LL), this feature has also been documented in several other *Pseudopoda* species (e.g., *P.exigua* (Fox, 1938)), and therefore does not conflict with its generic assignment.

Overall, the COI phylogenetic analyses provide robust molecular support for the generic placements of *P.tadungensis* sp. nov. and *H.taygiangensis* sp. nov., reinforcing the morphological diagnoses and underscoring the utility of integrative taxonomy in sparassid systematics.

## Supplementary Material

XML Treatment for Heteropoda taygiangensis

XML Treatment for Pseudopoda tadungensis

## ﻿Additional information

### Author ORCIDs

He Zhang https://orcid.org/0000-0002-1478-9837

Quang Duy Hoang https://orcid.org/0000-0003-1672-7960

Shuang Lei https://orcid.org/0000-0002-5732-5231

### Data availability

All of the data that support the findings of this study are available in the main text.

## References

[B1] Cao XW Liu J Chen J Zheng G Kuntner M Agnarsson I (2016) Rapid dissemination of taxonomic discoveries based on DNA barcoding and morphology. Scientific Reports 6(1): 37066. 10.1038/srep37066 27991489 PMC5171852

[B2] Folmer O Black M Hoeh W Lutz R Vrijenhoek R (1994) DNA primers for amplification of mitochondrial cytochrome c oxidase subunit I from diverse metazoan invertebrates. Molecular Marine Biology and Biotechnology 3: 294–299. 7881515

[B3] Jäger P (2008) Revision of the huntsman spider genus *Heteropoda* Latreille 1804: species with exceptional male palpal conformations from Southeast Asia and Australia (Arachnida, Araneae: Sparassidae: Heteropodinae). Senckenbergiana Biologica 88: 239–310. 10.11646/zootaxa.5352.1.1

[B4] Korai SK Jäger P (2024a) Five new species of *Heteropoda* Latreille, 1804 spiders (Araneae: Sparassidae) from Southeast Asia. Zootaxa 5481(2): 241–259. 10.11646/zootaxa.5481.2.4. 39646043

[B5] Korai SK Jäger P (2024b) Five new species of the spider genus *Heteropoda* Latreille, 1804 (Araneae: Sparassidae) from China. European Journal of Taxonomy 947: 109–129. 10.5852/ejt.2024.947.2623

[B6] Li JL Jäger P Liu J (2013) The female of *Heteropodaschwalbachorum* Jäger, 2008 (Araneae: Sparassidae). Zootaxa 3750(2): 185–188. 10.11646/zootaxa.3750.2.6 25113688

[B7] Logunov DV Jäger P (2015) Spiders from Vietnam (Arachnida: Aranei): new species and records. Russian Entomological Journal 24(4): 343–363. 10.15298/rusentj.24.4.09

[B8] Ono H Thinh TH Sac PD (2012) Spiders (Arachnida, Araneae) recorded from Vietnam, 1837–2011. Memoirs of the National Museum of Nature and Science Tokyo 48: 1–37.

[B9] Quan D Zhong Y Liu J (2014) Four *Pseudopoda* species (Araneae: Sparassidae) from southern China. Zootaxa 3754(5): 555–571. 10.11646/zootaxa.3754.5.2 24869707

[B10] Seyfulina RR Kartsev VM (2022) On the spider fauna of the Oriental Region: new data from Thailand (Arachnida: Aranei). Arthropoda Selecta 31(1): 115–128. 10.15298/arthsel.31.1.14

[B11] Simon E (1880) Révision de la famille des Sparassidae (Arachnides). Actes de la Société Linnéenne de Bordeaux 34(2/3/4): 223–351.

[B12] Simon E (1886) Arachnides recueillis par M. A. Pavie (sous chef du service des postes au Cambodge) dans le royaume de Siam, au Cambodge et en Cochinchine. Actes de la Société Linnéenne de Bordeaux 40: 137–166.

[B13] Sterling EJ Hurley MM (2005) Conserving Biodiversity in Vietnam: Applying Biogeography to Conservation Research. Proceedings of the California Academy of Sciences 56(9): 98–114.

[B14] World Spider Catalog (2025) World Spider Catalog. Version 26. Natural History Museum Bern. http://wsc.nmbe.ch [accessed on 1 April 2025]

[B15] Yamasaki T Henriques S Phung LTH Hoang QD (2018) Redescription of the sole species of the enigmatic solifuge genus *Dinorhax* Simon, 1879 (Solifugae: Melanoblossiidae) in Southeast Asia. The Journal of Arachnology 46(3): 498–506. 10.1636/JoA-S-17-090.1

[B16] Zhang H Zhong Y Zhu Y Agnarsson I Liu J (2021) A molecular phylogeny of the Chinese *Sinopoda* spiders (Sparassidae, Heteropodinae): implications for taxonomy. PeerJ 9: e11775. 10.7717/peerj.11775 PMC8381878 34484980

[B17] Zhang H Zhu Y Zhong Y Jäger P Liu J (2023) A taxonomic revision of the spider genus *Pseudopoda* Jäger, 2000 (Araneae: Sparassidae) from East, South and Southeast Asia. Megataxa 9(1): 1–304. 10.11646/megataxa.9.1.1

